# Reproductive Traits Demonstrate How Well the Mediterranean Stripe-Necked Turtle *Mauremys leprosa* Can Flourish under Highly Degraded–Polluted Conditions

**DOI:** 10.3390/biology11111562

**Published:** 2022-10-25

**Authors:** Sami Aymen Gahmous, Ghoulem Tiar, Manel Tiar-Saadi, Zihad Bouslama, Pavel Široký

**Affiliations:** 1 Ecology of Terrestrial and Aquatic Systems Laboratory, Faculty of Sciences, University Badji Mokhtar-Annaba, BP 12, El Hadjar, Annaba 23000, Algeria; 2 Functional and Evolutionary Ecology Laboratory, Faculty of Natural and Life Sciences, University Chadli Bendjedid-El Tarf, 76, El Tarf 36000, Algeria; 3 Environment and Biodiversity Research Division, National Environmental Research Center, Sidi Amar University Campus, 2024, Annaba 23005, Algeria; 4 National Environmental Research Center, Sidi Amar University Campus, 2024, Annaba 23005, Algeria; 5 Department of Biology and Wildlife Diseases, Faculty of Veterinary Hygiene and Ecology, University of Veterinary Sciences Brno, Palackého 1946/1, 612 42 Brno, Czech Republic; 6 CEITEC-Central European Institute of Technology, University of Veterinary Sciences Brno, Palackého 1946/1, 612 42 Brno, Czech Republic

**Keywords:** *Mauremys leprosa*, clutch size, egg dimension, female body size, reproductive trait, polluted habitat

## Abstract

**Simple Summary:**

The Mediterranean stripe-necked turtle *Mauremys leprosa* is known to possess physiological mechanisms allowing it to adapt without particular signs of physiological disorder or stress in habitats with high pollution. Nevertheless, we were uncertain about the costs of this adaptation and its impact on reproduction. The reproductive traits measured in this study suggest the adaptation of *M. leprosa* to flourish well in highly degraded–polluted areas compared to undisturbed habitats. *M. leprosa* was so well adapted to the pollution that the exposed population actually increased its reproductive capacity. Females living in the highly degraded–polluted habitat exhibited record body sizes for the species, which allowed them to carry both more and larger eggs. In comparison, the corresponding reproductive traits measured in the intact habitat ranged within the limits reported in previous studies.

**Abstract:**

We measured and compared the reproductive traits of the Mediterranean stripe-necked turtles *Mauremys leprosa* living in highly degraded–polluted vs. intact natural habitats in Algeria. Data on reproduction were obtained by using X-ray examination of gravid females and examination of nests. The results were opposite to the negative trend confirmed in most freshwater turtles exposed to pollution and suggested the ability of this species to flourish instead in highly degraded–polluted habitats. An optimum development was recorded for several reproductive patterns of the studied freshwater turtle under conditions considered uninhabitable for other vertebrates. Females exhibited record body sizes among conspecifics, which allowed them to carry significantly larger clutches, exceeding by up to 3 eggs the previously largest reported clutch. The mean clutch size (8.79 ± 2.70 eggs) was also higher than findings from previous studies, except for in some other polluted habitats. Furthermore, large females even with large clutches carried large eggs. Egg measurements in the disturbed habitat revealed new records exceeding those previously noted; in egg length (by 3.0 mm), egg width (by 2.8 mm), egg mass (by 1.8 g), and clutch mass (by 18.6 g). In comparison, the usual reproductive performances were observed in the intact natural habitat; female body sizes were significantly smaller and carried fewer eggs of smaller size.

## 1. Introduction

The Mediterranean stripe-necked turtle *Mauremys leprosa* (Schweigger, 1812) (Geoemydidae) is an endemic turtle to the western Mediterranean region, occupying all types of freshwater habitats, including rivers, intermittent streams, lakes, artificial reservoirs, and coastal marshes [1,2,3,4]. Its largest populations range over the Maghreb region, from Morocco to western Libya [5,6]. It occurs throughout northern Algeria in regions with a Mediterranean climate southwards penetrating the limits of the Sahara [6,7]. Outside the Maghreb, it is confined to the Iberian Peninsula [4,8,9] with some scarce populations spread in southern France [10]. Small introduced populations were reported in Italy and the Balearic Islands [11,12].

Data on life history, mainly on reproductive traits, are necessary to understand the biology of a species and to assess its adaptive mechanisms with regard to various anthropogenic disturbances, including pollution. *Mauremys leprosa* represents a relevant model for this issue. It is frequently recorded in polluted waters and tolerates muddy bottoms and eutrophic water habitats, which provide abundant food and limited competition, mainly by fish [2,13,14,15]. High densities were mentioned in highly degraded–polluted habitats, where individuals congregate in constrained spaces with limited movement, where the food supply is plentiful as the turtles take advantage of the organic waste, silt, and the macroinvertebrates [2,3,13,14,15].

Data on the reproductive biology of *M. leprosa* are scarce, especially from the Maghreb region. Two studies are available; the first pioneer study on the gonadal cycle was carried out in Algeria in the 1950s [16], while the second was conducted on sacrificed females in Morocco six decades later [17]. On the other hand, numerous studies on female reproduction and nesting pattern were carried out in the European part of the species’ range [18,19,20,21,22,23,24,25].

Recent studies have confirmed that this species can easily resist extremely degraded–polluted conditions without showing particular signs of ecological imbalance, physiological disorder, or stress [13,14,26,27]. El Hassani et al. [14] considered *M. leprosa* to be a biological indicator of high pollution tolerance, which is only equivalent among vertebrates to the brown rat *Rattus norvegicus*. To maintain an elevated metabolism and anabolism during reproduction, individuals in degraded–polluted habitats taking advantage of food abundance did not need to mobilize their body reserves, but rather accumulate them further and, consequently, produce larger body sizes than in undisturbed habitats [14,26]. However, other authors achieved contradictory conclusions regarding the accumulation capacity for toxic metals in polluted sites and its impact on the growth and immune response of exposed individuals [15,27,28]. Various effects, from lethal through endocrine disruption, genetic damage, to demographic changes, decreased energy devoted to reproduction, growth, and storage, were caused by various contaminants; organic, metallic, and radioisotopic contaminants have occurred in several turtle populations [27,28,29,30,31,32,33].

If the adaptation of *M. leprosa* populations to degraded habitats was fairly well demonstrated, the cost it may induce on its physiology, especially reproductive patterns remain uncertain. Will breeding female size, egg number, and size be affected? Three benchmark scenarios relating to the reproductive traits of the population studied could be envisaged: (i) the reproductive traits of the population could be completely affected by the environmental disturbance and exhibit a low reproductive performance; (ii) the environmental disturbance could be effectively reduced and turtles maintain normal performance around the mean values of the species’ range; or (iii) there would be an optimal status of reproductive traits towards upper thresholds, reflecting the flourishing of the population in high disturbance conditions. We aimed to assess the reproductive traits of *M. leprosa* in a situation of high pollution, as a proxy for new preferential habitats of the species in the Maghreb. In order to establish comparisons with a control habitat, we also studied a population living in an undisturbed and protected water body. The comparative data allow us to understand both the adaptive strategy selected by individuals living in a high level of degradation-pollution and the physiological cost, especially of reproduction, that the population has invested to persist in such a restrictive habitat.

## 2. Materials and Methods

### 2.1. Study Area

The fieldwork was conducted in two contrasting habitats, one highly degraded–polluted, the other undisturbed and monitored against toxic effluents. These are the estuary of Oued Boukhmira located in eastern Annaba province (36°50′48″ N; 7°48′50″ E), and Lake Tonga located in eastern El Kala province (36°51′37″ N; 8°29′52″ E), respectively. The two studied water bodies are part of the wetland complexes of the Mediterranean coast in the northeasternmost part of Algeria. The Boukhmira River receives various effluents, including from industrial, agricultural, and urban sources. Less than 4 km away, the river crosses a large wild dump of household waste, where it takes on significant quantities of organic waste but also pollutants of different natures. Domestic effluents and wastes from agriculture on the land adjacent to the estuary are another source of pollution. Lake Tonga is a large freshwater marsh of 2700 ha. It is part of El Kala National Park and is classified as both an integral reserve and a wetland of international importance in the Ramsar network. Therefore, the site is monitored for degradation, including pollution, thus maintaining the correct quality of its water and protecting the natural animal populations that inhabit it.

The samples in Boukhmira were taken from the intermediate and fluvial sections 2 km from the mouth of the estuary. The overall area of the nest site was 1 ha. Four turtle capture stations along the banks of the watercourse were established. At Lake Tonga, despite our regular surveys of the banks, we were unable to determine nesting sites and the samples were, therefore, only performed on turtles caught in the water body, with the same effort of four capture stations as at the first site.

The study areas were located on the warm sub-humid meso-Mediterranean bioclimatic floor, rainy in winter and hot and dry in summer [2]. The highest monthly mean temperatures are observed between June and September, ranging from 20 to 26 °C. The mean annual total rainfalls, according to the closest meteorological stations, were 641 mm in Annaba and 668 in El Kala [2].

The nesting site detected in Boukhmira is a wasteland abandoned by its former users more than 4 years ago. Its vegetation is dominated by *Verbascum sinuatum, Scolymus hispanicus,* and *Galectites tomentosus*. The river banks are completely covered by *Phragmites australis* and *Rubus fruticosus*. The vegetation composition of Lake Tonga was denser and more diversified, with emergent vegetation covering 90% of the water surface in spring and summer, dominated by *Phragmites australis*, *Scirpus lacustris*, *Typha angustifolia*, and *Iris pseudacorus*, in addition to submerged vegetation, represented by *Nymphaea alba* and *Potamogeton lucens* [34].

### 2.2. Field Procedures and Data Collection

The study period lasted over 6 months, between the beginning of April and the end of September of 2021. We took care to make sampling efforts in the two study habitats comparable. Two field visits per week per habitat were scheduled during the beginning of the spring, increasing to four field visits per week after the detection of the first gravid female, in order to increase our chances both of capturing other gravid females and of finding undestroyed nests. The females were captured by hand when they went out in search of a nesting site and using a net when they swam or basked. To distinguish recaptures, each turtle was individually marked on the marginal scutes of the carapace with a unique code following the method of Ernst et al. [35]. All captured turtles were sexed using external morphological criteria; only females were included in the study. Once captured, each female was palpated to check for the presence of eggs. Suspected gravid females were transported to the laboratory for radiography. An X-ray of constant 15 mA, 40 kV at 0.4 s distance was used according to Zuffi et al. [36]. The maximum egg length (EL) and maximum egg width (EW) of the X-rayed eggs were measured using software integrated into the X-ray equipment (±0.1 mm). The three measurements taken of the turtle shell (length, width, and height) were made using a caliper with accuracy 0.1 mm: straight midline carapace length (SCL; the straight anteroposterior distance between the nuchal and supracaudal scutes); mid-body carapace width (MCW; body width in the middle of the abdominal scute); and shell height (SH; the maximum vertical distance from the top of carapace to plastron). Following the sampling protocol, the females were released at the location of their captures.

Nests were detected by prospecting the banks of the river in search of nesting females. Nests were cautiously opened to count the eggs, which were carefully removed one by one. Each egg was subjected to three main measurements: maximum length (EL) and maximum width (EW) were measured with a manual caliper (50 mm ± 0.1 mm), and egg mass (EM) was determined using an electronic balance (82 g ± 0.01 g). We calculated individual clutch masses (CM) as the sum of egg masses within each clutch. The eggs were carefully returned to their original position after the measurements. The few accidentally broken eggs (circa 1.5% of the sampled eggs) were exempted from the sample and were not returned to the nest. We made sure to cover the nest and moisten it carefully to restore its original appearance.

### 2.3. Statistical Analyses

The mean ± standard deviation (SD) and range (min-max) were calculated for the morphometric descriptors of gravid females, eggs, and clutch size. In the descriptive statistics of egg measurements only data from nests were used; X-ray data were excluded. Analysis of variance (ANOVA) was performed to compare the measured biological traits between the two studied habitats, following testing for normality and homogeneity of distribution using the Kolmogorov–Smirnov and Levene’s tests, respectively. Correlations were calculated between the body size descriptors of gravid females (SCL, MCW, and SH) and their clutch sizes or with their egg sizes (EL and EW) calculated by radiography. Data were log-transformed for analysis where necessary. *p*-value of < 0.05 was considered to indicate a significant difference in all statistical tests. These statistical analyses were performed using Minitab Version 15.0 (Minitab, Inc., State College, PA, USA, https://www.minitab.com/en-us/), accessed on 1 July 2021).

## 3. Results

Despite the comparable sampling effort provided in both habitats, the number of females found gravid and detected intact nests was unbalanced. The number of sampled females was much higher in Boukhmira (346 individuals) compared to Lake Tonga (73 individuals). We were able to capture 43 gravid females in Boukhmira (12.4% of sampled females) and 12 in Lake Tonga (16.4%), all confirmed positive by radiography. The active efforts resulting from prospecting of the two sites resulted in the detection of 49 nests in Boukhmira, but none in Lake Tonga.

### 3.1. Body Size of Gravid Females

The one-way ANOVA comparison test of the body size descriptors carried out between the gravid females’ data from the two study sites revealed a significant difference of all three descriptors (SCL, MCW and SH), each of which was larger in Boukhmira (Table 1). The mean body lengths of females confirmed gravid by X-ray were 189.47 ± 11.41 mm in Boukhmira and 177.20 ± 11.45 mm in Lake Tonga. The smallest had lengths of 161.70 mm and 150.90 mm, respectively, while the largest were 213.4 mm and 190.3 mm, respectively. The average mid-body carapace width and shell height of females from Boukhmira were 133.74 ± 6.89 mm and 66.80 ± 5.54 mm, respectively, distinctly larger than females from Lake Tonga that were 121.26 ± 10.97 mm and 60.15 ± 5.97 mm, respectively (ranges in Table 1).

### 3.2. Clutch Size

**Figure 1:**
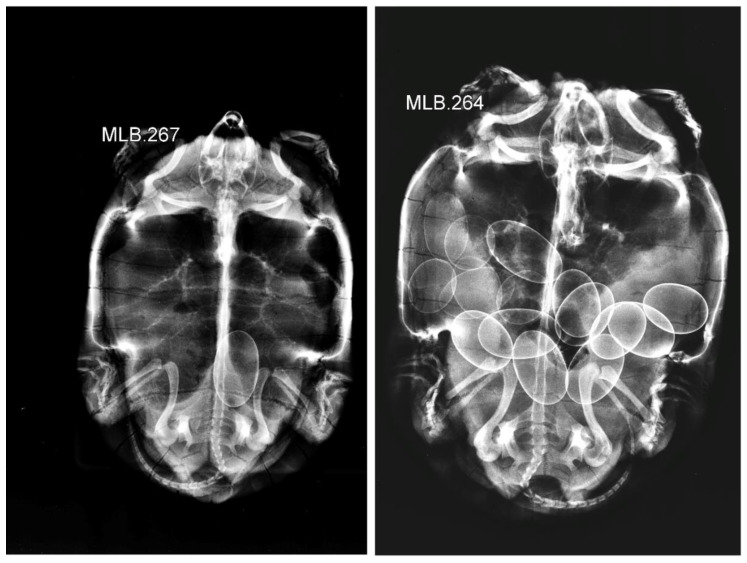
The two extreme clutch sizes (one egg and 16 eggs) detected in the Boukhmira population.

**Figure 2:**
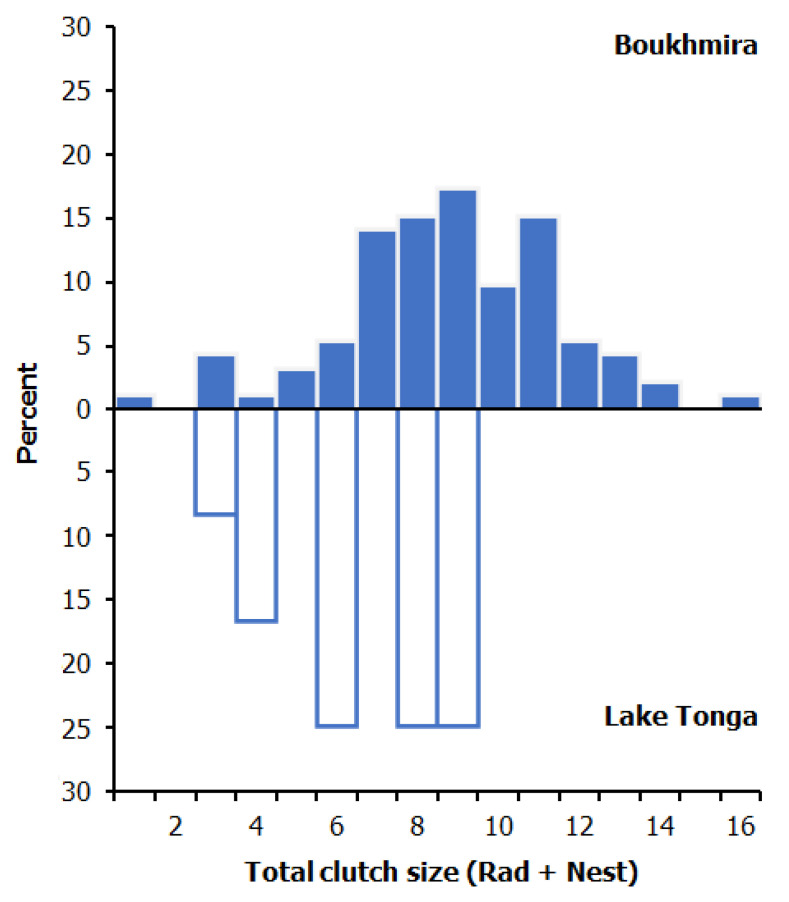
Distribution of clutch sizes in the studied populations, based on radiography and nesting data.

The average clutch sizes in the two studied populations based on x-rayed females were significantly different (ANOVA, site: *F*_(1, 53)_ = 15.14, *p* < 0.001), distinctly larger among the females of Boukhmira at 9.70 ± 2.45 eggs, than in Lake Tonga at 6.67 ± 2.15 eggs. Exclusively detected in Boukhmira, the nest clutch sizes when combined with the clutches obtained by X-ray gave an average clutch size of 8.79 ± 2.70 eggs (range, 1–16; *n* = 92). The two extreme clutches were detected by radiography, both observed in Boukhmira (Figure 1). The distribution of clutches showed a highly significant difference between the two studied populations (Figure 2). The majority of clutches in Boukhmira (77 %) consisted of 7–12 eggs, while the intervals of 1–6 eggs and 13–16 eggs represented 15% and 8% of clutches, respectively; however, the clutches from Lake Tonga did not exceed the size of 9 eggs and were predominately concentrated between 6 and 9 eggs (75% of clutches), and the remaining 25% ranged between 3 and 4 eggs. Descriptive statistics of clutch sizes based separately on nest counts, females’ radiography, or on a combination of both pooled datasets are summarized in Table 2.

### 3.3. Egg Sizes in Studied Populations

**Figure 3:**
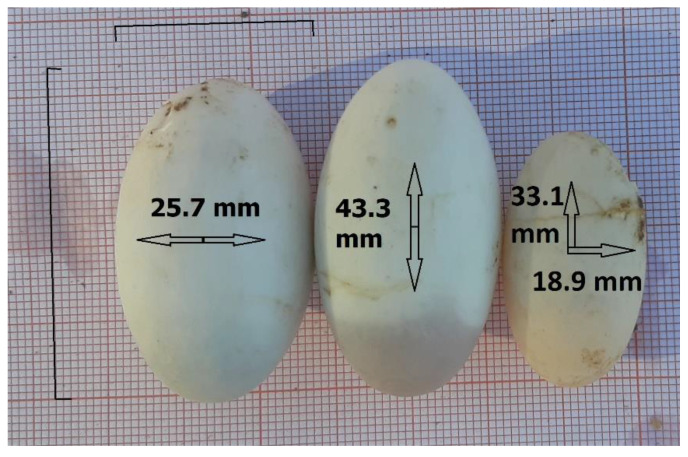
Demonstration of the morphometric differences between the widest (left egg), the longest (middle egg), and the egg close to the minimum dimensions (right egg) recorded during the present study.

Based on our methodological choice for the egg descriptive analysis of excluding the egg size measurements established on images by the X-ray software and relying on the direct measures made by caliper, only the population of Boukhmira provided data from nests. A total of 387 eggs were collected from 49 nests. The eggs had an average length of 35.95 ± 1.95 mm (31.8–43.3 mm) and an average width of 20.64 ± 1.17 mm (17.2–25.7 mm). The longest and widest eggs were photographed on graph paper (Figure 3). The mean egg mass (EM) and clutch mass (CM) were 8.69 ± 1.31 g (range: 4.7–12.5 g, *n* = 382) and 69.27 **±** 26.32 g (range: 21.0–127.5 g, *n* = 44), respectively.

The egg size measurements made on radiographic images did not show significant differences between the two studied populations (Mean-EL: *F*_(1, 53)_ = 0.09, *p* = 0.77; Mean-EW: *F*_(1, 53)_ = 0.05, *p* = 0.82).

### 3.4. Correlation of Clutch Size with Body Size of Gravid Females

**Figure 4:**
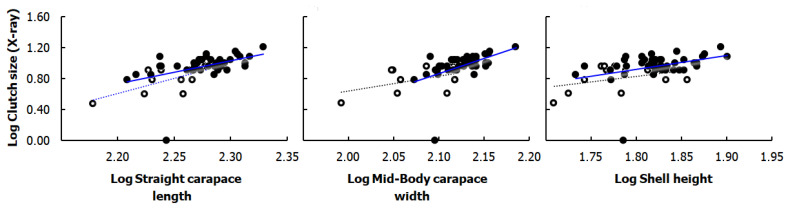
Linear regressions based on log-transformed data between selected body size measurements and clutch size obtained by X-ray in *Mauremys leprosa* from studied populations. Data from the Boukhmira population (the solid trendlines associated with the black circles) and the Lake Tonga population (the dotted trendlines associated with the open circles) were analyzed separately. Equations of significant regression lines (blue lines) are from the top left respectively: Log Clutch size = −5.86 + 3.00 Log SCL (R-Sq = 21.2%); Log Clutch size = −7.93 + 3.89 Log SCL (R-Sq = 49.1%); Log Clutch size = −7.45 + 3.96 Log MCW (R-Sq = 26.4%); Log Clutch size = −2.28 + 1.78 Log SH (R-Sq = 14.4%).

Clutch sizes were positively correlated with body length of gravid females in both studied habitats (SCL vs. Rad-CS Boukhmira: r = 0.57, *p* < 0.001, *n =* 43; Lake Tonga: r = 0.66, *p* < 0.05, *n =* 12); however, the correlations examined with mid-body width and shell height were significant in the Boukhmira population (MCW vs. Rad-CS, r = 0.66, *p* < 0.001, *n =* 43), (SH vs. Rad-CS, r = 0.47, *p* < 0.01, *n =* 43) but not at Lake Tonga (MCW vs. Rad-CS, r = 0.42, p = 0.18, *n =* 12), (SH vs. Rad-CS, r = 0.25, p = 0.43, *n =* 12) (Figure 4).

### 3.5. Relationships between Body Size of Gravid Females, Clutch Size, and Egg Size

**Figure 5:**
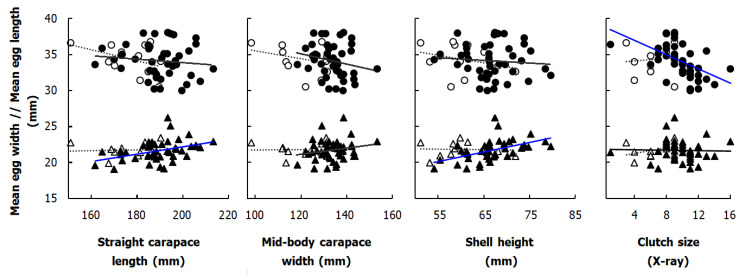
Linear regressions between both mean length (circles) and width (triangles) of eggs and three body size measurements in addition to the clutch size of *Mauremys leprosa* from the two studied populations (X-ray technique). The solid trendlines associated with the black-colored shapes represent the data of the Boukhmira population, while the dotted trendlines associated with the open shapes represent the data of Lake Tonga. Significant regression lines (in blue) are from the left, respectively: Mean EW = 12.11 + 0.05 SCL (R-Sq = 15.9%); Mean EW = 12.90 + 0.13 SH (R-Sq = 25.4%); Mean EL = 38.98 − 0.50 Rad-Clutch (R-Sq = 28.4%).

There was no significant correlation between female size descriptors and mean egg length in either habitat. The few significant correlations obtained came from data of the Boukhmira population, namely, mean egg width, which was positively correlated with SCL (r = 0.40, *p* < 0.01, *n =* 43) and with SH (r = 0.50, *p* < 0.01, *n =* 43) (Figure 5). Mean egg length was significantly negatively correlated to clutch sizes only in Boukhmira (Boukhmira: r = −0.53, *p* < 0.001, *n =* 43; Lake Tonga: r = 0.09, *p* = 0.78, *n =* 12), in contrast to mean egg width, which was not significantly correlated with clutch size in the two studied habitats (Boukhmira: r = −0.02, *p* = 0.893, *n =* 43; Lake Tonga: r = 0.42, *p* = 0.18, *n =* 12).

## 4. Discussion

The present study extends our knowledge about the impact of pollution on the reproductive traits of populations of the Mediterranean stripe-necked turtle. The obtained results in the highly degraded–polluted habitat are opposite to the negative trends reported in most freshwater turtles exposed to pollution, and conversely, an optimum development was recorded for several reproductive patterns, in comparison to a usual reproductive performance in the undisturbed habitat.

### 4.1. Body Size of Gravid Females

The difference in the sample sizes obtained in the two habitats does not prevent drawing satisfying comparisons. The number of sampled females was much higher in Boukhmira than in Lake Tonga, which may be due to the difference in their population densities. Tiar-Saadi [2] estimated the Boukhmira population density 23.9 ± 11.8 individuals/ha, one and a half times denser than Lake Tonga, 9.1 ± 11.3 individuals/ha.

The large body sizes of gravid females achieved in the highly degraded–polluted habitat reflect a reproductive performance pulled towards optimal limits, thus suggesting a strong evolutionary selection advantageous to large maternal sizes. The minimum body length of a gravid female, commonly referred to as the effective maternal size at maturity, varies in the literature from 124 mm to 153 mm [16,17,18,19,20,23]. Comparing to this range, the corresponding values of the two studied populations were divergent; within the usual range in the protected habitat, but higher than the range that can be considered as the largest size at maturity in the highly degraded–polluted habitat.

Similarly, referring to the mean body size data recorded among gravid females, the population of the highly degraded–polluted habitat provided the largest value recorded so far [17,19,20]. The female with largest clutch size was the largest gravid female not only in the studied populations but also compared to the data in the literature [16,17,18,19,20,23]. The only exception was recorded in the Spanish population in Doñana National Park, which contained a larger gravid female that nevertheless, carried a smaller clutch size [21]. 

This finding of favored larger body size in eutrophic and polluted conditions is not the most frequent; however, it is the dominant tendency in some generalist species of freshwater turtles, such as congeneric *Mauremys rivulata*, but also in *Actinemys marmorata, Chrysemys picta, Trachemys scripta*, and *Phrynops geoffroanus* [29,37,38,39,40,41]. These species reach larger body sizes in polluted habitats. On the contrary, several specialist species show unbalanced development when exposed to pollution [29,30,31,32,33].

Phenotypic variations in size at maturity may be due to the genetic difference of the studied populations; however, we can exclude the taxonomic involvement hypothesis in our results. Data on conspecifics mentioned in some earlier articles come from populations belonging to different subspecies of *M. leprosa* [42,43]. Our sampled populations belong to subspecies *M. l. saharica* as suggested for all northern Algerian populations [42,44]. It is the dominant taxon throughout North Africa, except for a few limited populations from the northern Atlas Mountains in Morocco. Recent studies recognize two clades of *M. leprosa*, one corresponding to the nominal subspecies *M. l. leprosa* distributed across the Iberian Peninsula, southern France and the mentioned Moroccan populations and the second (*M. l. saharica*) confined to north Africa, i.e., northern and southern Moroccan Atlas Mountains, eastern Algeria, and northern Tunisia [42,43,44]. Moreover, according to several studies, populations of the same taxa may also differ in their sizes at maturity and their growth when subjected to different environmental pressures [45,46,47]. The most involved biotic factors are variations in trophic resources [48,49], competition, and predation [50,51].

We are increasingly convinced of the physiological predisposition of the species to effectively resist negative impacts of high levels of pollution. Several recent studies have reported the resistance of *M. leprosa* and even flourishing of its populations in sites receiving different kinds of pollution (industrial, domestic, and agricultural) that we can connect with our study site [13,14,26,27]. El Hassani et al. [14] hypothesized the efficient response of *M. leprosa* to extremely polluted water bodies (the sewers of a large city amidst a huge open dump) as reduced metabolic and hormonal investment and no particular sign of physiological disorder or stress, qualifying it to be a biological model of high pollution tolerance. Martínez-López et al. [27] hypothesized that *M. leprosa* exhibits high effectiveness of the physiological mechanism of detoxification, allowing this species to adapt without consistent cost in habitats of high industrial pollution uninhabitable for other vertebrates.

Enjoying the above described ability to adapt to a restrictive environment, the species monopolizes the resources of the water body and its supplies, taking advantage of the disappearance of competitors and predators, which have no comparable performance [13,14,17]. Jorgewich-Cohen et al. [49] strongly implicated ecological factors, mainly resource availability on clutch capacity and egg size of chelonian species. The very wide food spectrum of *M. leprosa* (omnivory with a predominance of carnivory, but also scavenging and coprophagy) allows it to obtain supplies at will in eutrophic and polluted waters [8,13,52]. In Boukhmira, several groups of turtles consisting of a few dozen individuals were frequently noted eating the abundant aquatic invertebrates, rushing toward the organic waste freshly thrown by the local inhabitants, and also feeding on the corpses of fish and other vertebrates found on the surface of the stream (Pers. Obs.). Its enrichment with organic matter resulting from urbanization contributes to the productivity of aquatic invertebrates but, at the same time, to the reduction of their potential predators, who are unable to withstand the high levels of pollutants. High organic matter can also cause temporarily very low oxygen levels, which should not directly impact the turtles but would impact animals respiring with gills. Nevertheless, *M. leprosa* succeeds where the vast majority of vertebrates fail, including the other native freshwater turtle *Emys orbicularis* [2,7]. The costs of pollution were rewarded with food abundance to many hungry females, allowing them to invest considerably in egg production.

### 4.2. Clutch Size 

The mean clutch size recorded in the population of the highly degraded–polluted habitat exceeds most clutch size records among conspecific populations, usually ranging between 3.2 and 6.9 eggs (range 3–9), mainly from the natural sites of the Doñana National Park in Spain [18,20,21,23], and from an Algerian population, with 4.8 eggs (range 4–6) [16]. The natural population of Lake Tonga recorded a clutch size within the range of values in the literature. In contrast, the only data that presented higher values than Boukhmira, of 9.6 and 9.7 eggs (range, 3–13), come from populations occurring in polluted habitats [17,19].

Only a single population among five with unpolluted habitats in Doñana National Park reached a clutch size of 13 eggs [21]; the others do not have clutches of more than 10 eggs [18,20,21,23]. In the present study, one of the x-rayed gravid females carried 16 eggs, 3 eggs more than the previous maximum recorded [17,19]. This reproductive performance recorded in a highly degraded–polluted habitat seems not to be exceptional, because 8% of gravid females and sampled nests contained 13 or more eggs. Larger clutch size in lagoons receiving waste compared to a population inhabiting pristine habitats was observed also in *Chrysemys picta* [38]. Nevertheless, a larger number of studies on populations living in polluted water bodies have shown that both turtles and their eggs were contaminated with toxic substances, thereby influencing their reproductive potential [53,54,55,56].

### 4.3. Relationships between Female Body Size, Clutch and Egg Size

The selection for greater reproductive success and greater fecundity exercised by mature males, resulting in large female sizes is well documented in the literature [45,46,57,58]. Natural selection for greater fecundity supports larger females having greater reproductive capacity; a larger body accommodates larger clutches, larger eggs, or larger annual egg production [58,59,60]. In several turtle studies, the female body sizes and all three dimensions, length, width, and height were correlated with their fecundity [36,45,59]. Likewise, in the present study, clutch size was significantly correlated with female body size. Similar findings on the same species were established in previous studies [17,19]. We also recorded a positive correlation between female body sizes and their egg dimensions, especially the correlation of egg width with body length and with shell height, similar to some earlier studies on *M. leprosa* [17,18,19]. Otherwise, the non-significance of some correlations established at Lake Tonga could be explained by the small sample size from this habitat.

Moreover, in the highly degraded–polluted habitat, egg length was constrained by clutch size, since they were negatively correlated. This result is consistent with the predictions from optimal egg and clutch size theory, which assume that large-bodied turtles tend to produce larger clutches with small eggs [49,60,61]. However, the correlations in the Spanish conspecific populations, as in the undisturbed habitat population of the present study, were not statistically significant [18,19].

The egg size recorded in the present study exceeds the measurement previously noted in Spain [19,24]. This holds the same for the measurement of the mean egg length, which was a little longer than the previous record value [18]. On the contrary, the obtained mean egg width was comparable to the available data [16,17,18,19,20,24].

The larger dimensions of the sampled eggs obtained in the highly degraded–polluted habitat generated larger weights. Three record traits were observed compared to the previously published studies, namely, the heaviest egg weighed 1.8 g more than the maximum recorded value; the mean egg mass exceeded the reported maximum by 0.4 g; and the clutch mass was 18.6 g higher than the previously published record value [17,19]. Similar to the evolutionary mechanisms on clutch size explained above, egg size and egg mass constitute a selective advantage for increasing the fitness of the species, because large eggs produce large hatchlings, which usually have increased performance and survivorship [59,60,61].

## 5. Conclusions

*Mauremys leprosa* is a species with a large tolerance for ecological variations, which survives and reproduces in many sites under variable conditions. In the Maghreb, it is very common in a wide range of aquatic habitats, from unspoiled natural streams to very disturbed sewers of poor quality. This study allowed us to appreciate the adaptation of the reproductive characteristics of a species in an environment believed to be uninhabitable for vertebrates and its successful survival due to the effectiveness of its physiological mechanisms. Such ability to adapt to high levels of pollution, without stress or significant physiological cost, has resulted in the optimum development of its reproductive performance, benefiting from trophic abundance and taking advantage of the absence of potential competitors or predators, which cannot tolerate such conditions.

However, considering the enlargement of anthropogenic environments and the accentuation of various discharges and effluents into water bodies in the Maghreb in recent years, it is likely that *M. leprosa* will further spread its populations and distribution. It could be undesirable if *M. leprosa* expand to the detriment of other local aquatic vertebrates, particularly the only sympatric native freshwater turtle species *E. orbicularis*, which on the contrary, seems to be in decline. The collection of its distribution data in Algeria and all of the Maghreb, coupled with the habitat properties, including levels of toxins such as DDT, Hg, or hydrocarbons, will provide us with an essential tool to guide future conservation strategies and/or action plans.

## Figures and Tables

**Table 1:** Morphometric data of sampled gravid females in the two studied habitats. Var: variable; SD: standard deviation; *n* = sample size; SCL: straight midline carapace length; MCW: mid-body carapace width; SH: shell height.

Scheme.	Boukhmira			Lake Tonga			
Var. (mm)	Mean ± SD (*n* = 43)	Smallest Gravid Size (mm)	Largest Gravid Size (mm)	Mean ± SD (*n* = 12)	Smallest Gravid Size (mm)	Largest Gravid Size (mm)	ANOVA Test
SCL	189.47 ± 11.41	161.70	213.40	177.20 ± 11.45	150.90	190.30	F_(1, 53)_ = 10.85, *p* < 0.01
MCW	133.74 ± 6.89	118.40	153.30	121.26 ± 10.97	98.30	131.40	F_(1, 53)_ = 23.36, *p* < 0.001
SH	66.80 ± 5.54	54.10	79.60	60.15 ± 5.97	51.20	71.90	F_(1, 53)_ = 13.05, *p* < 0.001

**Table 2:** Clutch size descriptive statistics of the studied populations. Var: variable; SD: standard deviation, *n*: sample size, N-CS: clutch size sampled in nests, RAD-CS: clutch size detected in females by radiography, Total-CS: pooled clutch size obtained by combination of both nest counts and x-rayed females.

Site	Boukhmira			Lake Tonga		
Var.	Mean ± SD	Range	*n*	Mean ± SD	Range	*n*
N-CS	8.00 ± 2.68	3–14	49	-	-	-
RAD-CS	9.70 ± 2.45	1–16	43	6.67 ± 2.15	3–9	12
Total-CS	8.79 ± 2.70	1–16	92	6.67 ± 2.15	3–9	12

## Data Availability

The data presented in this study are available on request from the corresponding author. The data are not publicly available because the authors will use them in future research.
